# Ginsenoside-Rb1-Mediated Anti-angiogenesis via Regulating PEDF and miR-33a through the Activation of PPAR-γ Pathway

**DOI:** 10.3389/fphar.2017.00783

**Published:** 2017-11-13

**Authors:** Huixia Lu, Xunian Zhou, Hoi-Hin Kwok, Mei Dong, Zhaoqiang Liu, Po-Ying Poon, Xiaorong Luan, Ricky Ngok-Shun Wong

**Affiliations:** ^1^Key Laboratory of Cardiovascular Remodeling and Function Research, Chinese Ministry of Education and Chinese Ministry of Health, State and Shandong Province Joint Key Laboratory of Translational Cardiovascular Medicine, Department of Cardiology, Qilu Hospital of Shandong University, Jinan, China; ^2^Gilbert Hung Ginseng Laboratory, Department of Biology, Faculty of Science, Hong Kong Baptist University, Hong Kong, Hong Kong; ^3^Department of Ophthalmology, Shandong Provincial Hospital affiliated to Shandong University, Jinan, China

**Keywords:** ginsenoside Rb1, angiogenesis, miR-33a, PEDF, peroxisome proliferator-activated receptor-γ (PPAR-γ)

## Abstract

Angiogenesis is the formation of new blood vessels from the existing vasculature, which is involved in multiple biological processes, including atherosclerosis, ischemic heart disease, and cancer. Ginsenoside-Rb1 (Rb1), the most abundant ginsenoside isolated form Panax ginseng, has been identified as a promising anti-angiogenic agent via the up-regulation of PEDF. However, the underlying molecular mechanisms still unknown. In the present study, human umbilical vein endothelial cells (HUVECs) were selected to perform *in vitro* assays. Rb1 (0–20 nM) treatment induced pigment epithelial-derived factor (PEDF) protein expression in concentration and time-dependent manners. Interestingly, it was also demonstrated that the exposure of Rb1 (10 nM) could increase PEDF protein expression without any alteration on mRNA level, suggesting the involvement of posttranscriptional regulation. Furthermore, bioinformatics predictions indicated the regulation of miR-33a on PEDF mRNA 3′-UTR, which was further confirmed by luciferase reporter gene assay and real-time PCR. Over-expression of pre-miR-33a was found to regress partly Rb1-mediated PEDF increment and anti-angiogenic effect in HUVECs. Additionally, Rb1-reduced miR-33a and increased PEDF expression was prevented by pre-incubation with peroxisome proliferator-activated receptor-γ (PPAR-γ) antagonist (GW9662) or transfection with PPAR-γ siRNA in HUVECs. Taken together, our findings demonstrated that Rb1 exerted anti-angiogenic effects through PPAR-γ signaling pathway via modulating miR-33a and PEDF expressions. Thus, Rb1 may have the potential of being developed as an anti-angiogenic agent, however, further appropriate studies are warranted to evaluate the effect *in vivo*.

## Introduction

Angiogenesis is the development of new vessels from the pre-existing microvesseles, which plays a vital role in a variety of physiological and pathological processes, such as atherosclerosis, diabetic retinopathy, age-related macular degeneration, tumor growth, and metastasis, etc. (D'Alessio et al., 2015; Secomb and Pries, 2016). Angiogenesis is initiated by the degradation of basal membrane, which contributes to the enhanced vascular permeability and more dissolution of extracellular matrix, followed by endothelial cell migration, proliferation. All of these steps finally resulted in the formation of associated pericytes and leading to the increased new blood vessel formation.

Ginseng, a well-known traditional Chinese medicine, has been considered an “all healing” panacea for a long time. Most of the pharmacological activity of ginseng is attributed to its constituents, namely ginsenosides. On the basis of their aglycones, ginsenosides are classified structurally into protopanaxadiol (PPD)-type and protopanaxatriol (PPT)-type (Yue et al., 2007). Ginsenoside Rb1 and Rg1 are representative PPD- and PPT-type saponins, respectively. The “Yin and Yang” action of ginseng on angiomodulation is mainly decided by the compositional ratio between Rg1 and Rb1 (Yue et al., 2007). Rb1 is a representative component of ginseng possesses several properties, including anti-obesity, anti-oxidative stress, and anti-fatigue, anti-angiogenesis etc. (Sengupta et al., 2004; Cheng et al., 2005; Xiong et al., 2010). Additionally, Rb1 and its metabolite have been demonstrated to suppress angiogenesis in tumor cells (Sato et al., 1994; Shibata, 2001; Kim et al., 2004). Our previous findings had also suggested that Rb1 was able to inhibit angiogenesis through the up-regulation of pigment epithelial-derived factor (PEDF) in HUVECs (Leung et al., 2007).

PEDF, originally discovered from the retinal pigmented epithelium, has been recognized as the most potent natural angiogenesis inhibitor (Dawson et al., 1999). It plays a vital role in maintaining the avascular nature of the cornea and determining the balance between angiogenesis and anti-angiogenesis. Previous studies have found that PEDF exerted angiogenesis inhibitory effect in several disorders, such as ocular diseases, cardiovascular diseases, and cancer, etc. (Takenaka et al., 2008; Tombran-Tink, 2010; Becerra and Notario, 2013).

MicroRNAs (miRNAs) are tiny, endogenously expressed non-coding RNAs (18–25 nt long). They regulate crucial posttranscriptional mRNA expression, typically by binding to the 3′ untranslated region (3′-UTR) of the complementary mRNA sequence, thereby repressing translation and genes silencing (Wen, 2016). Recent studies have identified that miRNAs are highly expressed in endothelial cells (ECs), which are also believed to be novel regulators for the regulation of the vascular development and angiogenesis (Zhang, 2010). Moreover, modulation of miRNAs levels may also have potential therapeutic function for the treatment of angiogenesis related diseases (ARD), such as heart disease, cancer, and neurodegeneration, etc. (Andreou et al., 2015).

In the present study, the potential role of miR-33a in the Rb1-induced PEDF expression and the functional role of miRNA in anti-angiogenic action had been explored. This is the first study revealing that Rb1 exerted anti-angiogenic effects via the modulation of miR-33a and its target gene PEDF expression through PPAR-γ signaling.

## Materials and methods

Ginsenoside-Rb1 with ~98% purity was obtained from Fleton (Chengdu, China) (Kwok et al., 2012). Cell culture medium M199 was purchased from Invitrogen (Carlsbad, CA, USA). Fetal bovine serum (FBS), penicillin–streptomycin (PS), phosphate-buffered saline (PBS), and trypan blue were purchased from Invitrogen (Carlsbad, CA, USA). Endothelial cell growth supplement (ECGS) was from Upstate (Waltham, MA, USA). Anti-PEDF antibody was obtained from R&D Systems (Minneapolis, MN, USA), anti-GAPDH antibody was from Santa Cruz Biotechnology (California, CA, USA). Horseradish peroxidase-conjugated (HRP)-conjugated secondary antibodies were purchased from Zymed (South San Francisco, CA, USA). Lipofectamine®2000 and Opti-MEM® reduced serum were purchased from Life Technologies (Carlsbad, CA, USA). MiRNA precursor and negative control were from Applied Biosystems (Foster City, CA, USA). All the inhibitors were bought from Tocris Bioscience (Atlantic Road, Bristol, UK). PPAR-α siRNA and PPAR-γ siRNA were obtained from Ambion (Austin, Texas, USA).

### Cell culture and treatments

HUVECs (Lonza, Walkersville, MD, USA) were cultured in 1% gelatin-coated flasks with M199 containing 20% heat-inactivated fetal bovine serum (HI-FBS). HUVECs were then adapted in phenol red-free medium (PRFM) supplemented with 20% charcoal/dextran-treated FBS (CT-FBS, for removal of endogenous steroids in the serum) for 24 h and starved for overnight in PRFM with 1% CT-FBS before each assay. In general, cells were used between passages 2 and 8. Culture medium with DMSO (1%) served as the control group. HUVECs at 70% confluence were transiently transfected with miRNA precursor (pre-miR-33a, 50 nM) in parallel with the miRNA precursor negative control (pre-control, 50 nM). Transfection of pre-miR-33a or pre-control involved the use of Lipofectamine®2000 according to the manufacturer's protocol. PPAR-α siRNA (40 nM) and PPAR-γ siRNA (40 nM) were used for transient transfection of HUVECs for 24 h, followed by Rb1 treatment for 24 h. For screening the potential nuclear receptors mediating the effects of Rb1, cells were pre-treated with estrogen receptor (ER) antagonist ICI 182,780 (ICI, 20 μM), glucocorticoid receptor (GR) antagonist (RU486, 5 μM), PPAR-α antagonist (GW6471, 10 μM), PPAR-β antagonist (GSK0660, 10 μM), or PPAR-γ antagonist (GW9662, 10 μM) for 30 min at 37°C before Rb1 stimulation.

### Western blot analysis

Total protein from HUVECs lysates was collected after appropriate treatment. Protein concentrations were determined by Bradford protein assay (Bio-Rad Laboratories, Munchen, Germany) with bovine serum albumin as a standard. Equally loaded protein samples were subjected to SDS-PAGE and then transferred onto nitrocellulose membranes, which were incubated with PEDF antibody (1:250, 07-280, Upstate, and 1:250, AF1177) and GAPDH (1:1,000, sc20357) overnight at 4°C. Membranes were then incubated with horseradish peroxidase-labeled secondary antibodies (dilution 1:1,000) for 1 h at room temperature. The intensity of protein bands was determined by densitometry. Protein expression was assessed relative to GAPDH expression and expressed as a ratio. Image J software was used for the quantification of the protein bands in western blots.

### Quantitative real-time PCR (qRT–PCR)

Total RNA was extracted by using TRIzol (Invitrogen) according to the manufacturer's protocol. cDNA was synthesized by using the Superscript III first-strand synthesis system (Invitrogen) and mRNA level was quantified with the Kapa SYBR FAST qPCR Master Mix (Kapa Biosystems, Wilmington, MA, USA) and Mx3000P real-time PCR machine (Stratagene). The PCR mix was denatured at 95°C for 3 min, followed by 40 cycles of melting at 95°C for 3 s and annealing at 60°C for 20 s. The 2^−ΔΔCt^ method was used to calculate relative expression of the target gene, which was normalized to the internal control GAPDH. For miRNA measurement, TaqMan MicroRNA reverse transcription stem-loop primers were used according to the manufacturer's protocol and all real-time PCR reactions were determined on a StepOnePlus PCR system. The PCR mix was denatured at 95°C for 10 min, followed by 40 cycles of melting at 95°C for 15 s and annealing at 60°C for 60 s (Kwok et al., 2017). The internal control U6 was determined in parallel and used to normalize the relative expression of miR-33a expression. Primers are shown in Table 1.

**Table 1:** Primers used for real-time PCR amplifications.

**Name**	**Sequence (5′-3′)**
hPPARα F'	ACTCCACCTGCAGAGCAACCA
hPPARα R'	TAGATCTCCTGCAGTAGCGGG
hPPARγ F'	TGCACTGGAATTAGATGACAGC
hPPARγ R'	TCCGTGACAATCTGTCTGAGG
PEDF F'	TCACAGGCAAACCCATCAAGCTGAC
PEDF R'	GCCTTCGTGTCCTGTGGAATCTGCT
GAPDH F'	ATCAGCAATGCCTCCTGCAC
GAPDH R'	TGGTCATGAGTCCTTCACG

### Luciferase reporter gene assay

The 3′-UTR of SERPINF1 was cloned into the pLightSwitch-3′-UTR vector (WT) (SwitchGear Genomics, Menlo Park, CA, USA). To confirm the predicted binding site, construct with mutated complementary sequence to the seed region of miR-33a (MUT) were generated by the QuickChange Lightning Multi Site-Directed Mutagenesis Kit (Agilent Technologies, Palo Alto, CA, USA). Mutation was confirmed by DNA sequencing. COS-7 cells were cultured in DMEM supplemented with 10% FBS at 37°C in 5% CO_2_. Seeded cells were co-transfected with the SERPINF1 3′-UTR reporter construct (50 ng/well), SV40-driven firefly luciferase reporter (0.1 ng/well) (Promega, Madison, WI, USA), and miR-33a synthetic oligonucleotides (50 nM) by Lipofectamine®2000 (Invitrogen) for 24 h, then lysed with lysis buffer, and luciferase activity was measured by use of the Dual Luciferase Reporter Assay System (Promega) with a microplate luminometer (Infinite F200; Tecan, Männedorf, Switzerland). The relative luciferase activity was normalized to firefly luciferase activity.

### Tube formation assay

HUVECs were seeded in the 24-well plate coated with growth factor-reduced matrigel. Cells were transfected with pre-miR-33a or pre-control in the presence or absence of Rb1 for 13–15 h. Images were captured and 12 microscopic fields were randomly selected for each well. The number of tubelike structures per field was counted. Then the value of control group was normalized to 100% for further calculation of the relative percentage of tube formation.

### Statistical analysis

All experiments were repeated at least three times, and data are presented as mean ± S.EM. Statistical analysis involved one-way ANOVA followed by LSD *post-hoc*-test (SPSS version 21, San Diego, CA, USA). Statistical comparisons between two groups involved Student's *t*-test. *P* < 0.05 was considered statistically significant.

## Results

### *Rb1* induced *PEDF* expression and diminished *miR-33a* expression in *HUVECs*

**Figure 1:**
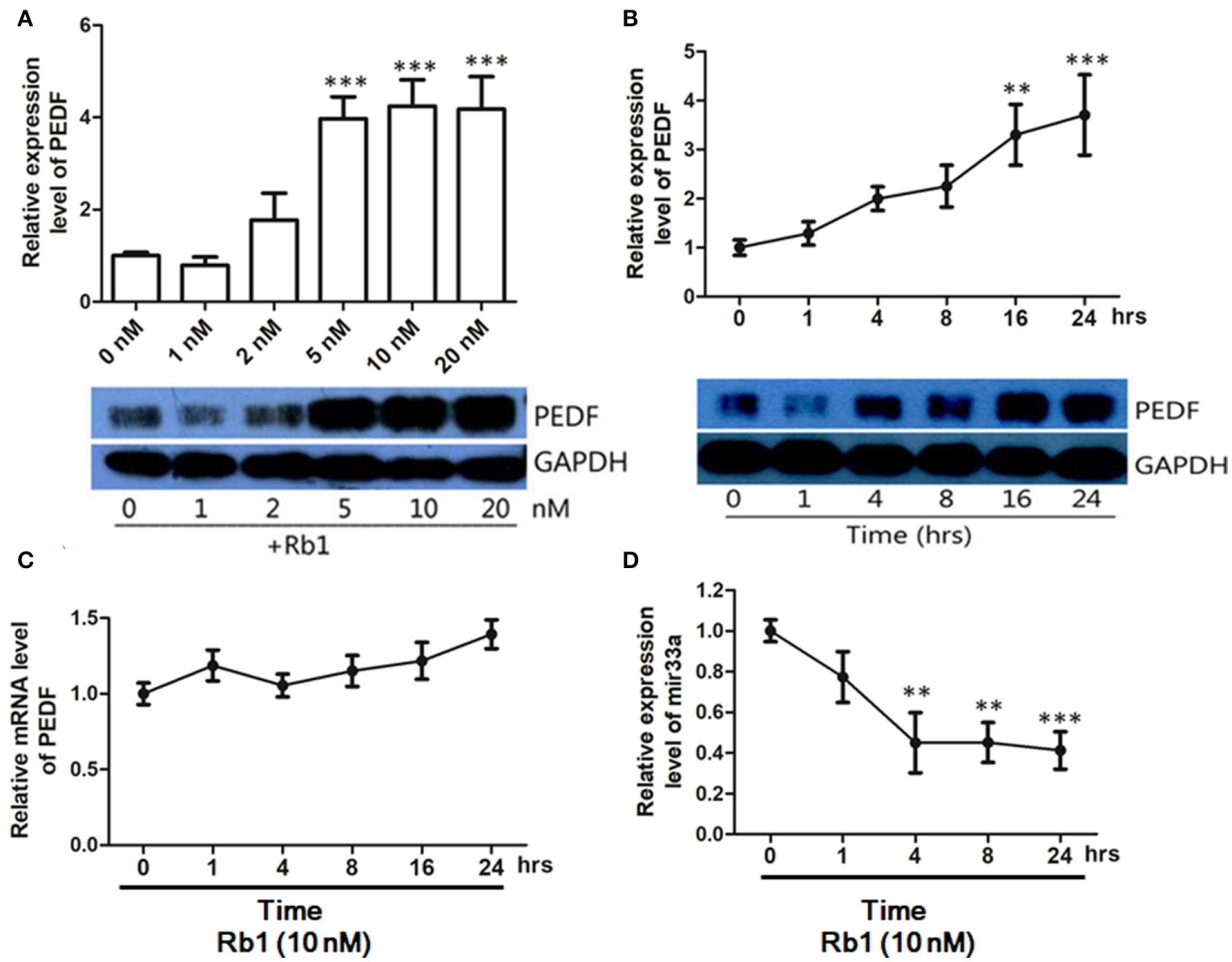
PEDF protein and mRNA expression in HUVECs treated with Ginsenoside Rb1. **(A)** Western blot analysis and quantification of PEDF expression in HUVECs treated with different concentrations of Rb1. Culture medium with DMSO (1%) served as the control group. **(B)** Western blot analysis and quantification of PEDF expression in HUVECs treated with 10nM Rb1 at different timepoints. **(C)** PEDF mRNA expression in HUVECs treated with 10 nM of Rb1 at different timepoints. **(D)** MicroRNA-33a expression levels in HUVECs treated with 10nM Rb1 at different timepoints. The relative expression of miR-33a was calculated against U6 RNA using the comparative Ct method (2^−ΔΔCt^). Each value was expressed as fold of control mean + S.EM. (*n* = 3). ^**^vs. con *p* < 0.01; ^***^vs. con *p* < 0.001.

PEDF is a potent anti-angiogenic factor. To determine the effect of Rb1 on PEDF expression, HUVECs were treated with different concentrations of Rb1 for 24 h. PEDF protein levels were upregulated from 5 to 20 nM (Figure 1A). We selected 10 nM Rb1 in the following experiments. Rb1 (10 nM) could time-dependently increase PEDF protein expression (Figure 1B). However, PEDF mRNA level was unchanged in HUVECs with 10 nM Rb1 (Figure 1C). To further understand how Rb1 regulates PEDF, miRNA microarray assay (Beijing Genomics Institute, Shenzhen, China) revealed a series of miRNA candidates significantly changed in expression in HUVECs after Rb1 (10 nM) treatment (data not shown). By combining the prediction results of three computational target prediction programs (TargetScan, microcosm, and PicTar), we predicted six putative miRNAs (miR-33a, 33b, 187, 486-3p, 586, 600) that target PEDF. It was identified that miR-33a level was significantly changed (*p* < 0.0001, fold change > 2) from miRNA microarray result. In addition, real-time PCR was applied to characterize the change in expression of these miRNAs. Rb1 (10 nM) treatment for 4 h could significantly decrease approximately half of miR-33a expression when normalized to the U6 control, and such suppression remained stable till 24 h (Figure 1D).

The changes in other putative miRNA expressions are not shown here.

### Identification of *PEDF* as a target gene of *miR-33a* in *HUVECs*

**Figure 2:**
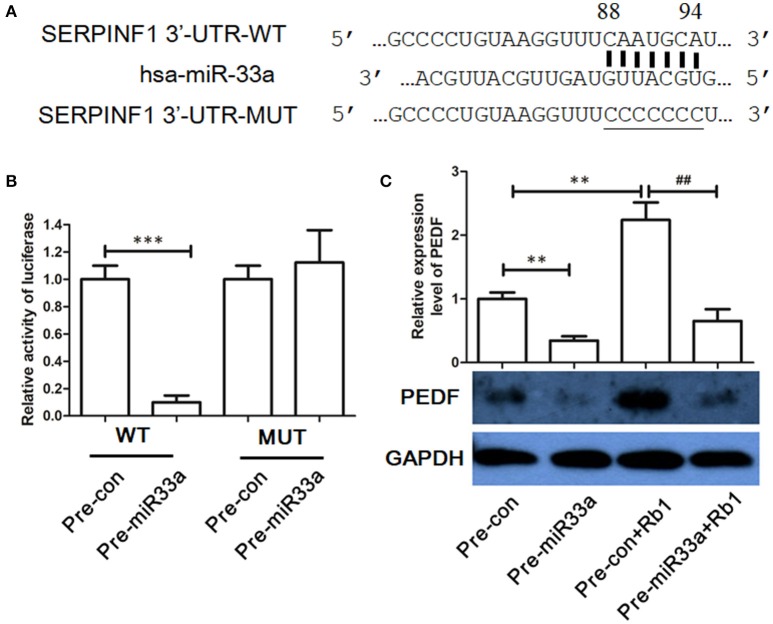
Identification of PEDF as target gene of miR-33a. **(A)** The predicted binding site in the 3′-UTR of PEDF (SERPINF1) and the mutated region of the predicted binding site. **(B)** miR-33a mimic (50 nM) or nontargeting control (50 nM) was cotransfected with the luciferase reporter carrying wild type-PEDF 3′-UTR or mutated PEDF 3′-UTR. Twenty-four hours after transfection, luciferase activities were measured (*n* = 3). **(C)** Western blot analysis and quantification of PEDF expression in Rb1-treated HUVECs with or without miR-33a transfection. Each value was expressed as fold of control mean + S.EM. (*n* = 3). ^**^vs. con *p* < 0.01; ^***^vs. con *p* < 0.001; ^*##*^vs. Rb1 treatment alone *p* < 0.01.

To determine whether the 3′-UTR of PEDF was the direct binding site of miR-33a, we used luciferase reporter assay. Overexpression of miR-33a but not the control miRNA substantially repressed the luciferase activity with WT-3′-UTR of PEDF (Figure 2). In contrast, no effect was found when the miR-33a-binding sites in the 3′-UTR of PEDF were mutated (Figures 2A,B), which suggested that miR-33a can directly bind to the 3′-UTR of PEDF and regulate its expression. To further determine the role of miR-33a in Rb1-induced PEDF expression, pre-miR-33a was transfected into HUVECs with or without Rb1 treatment. Rb1-enhanced PEDF expression was significantly reversed by the transfection of pre-miR-33a in HUVECs (Figure 2C).

### *miR*-*33a* is a novel mediator in anti-angiogenic action of *Rb1* in *HUVECs*

**Figure 3:**
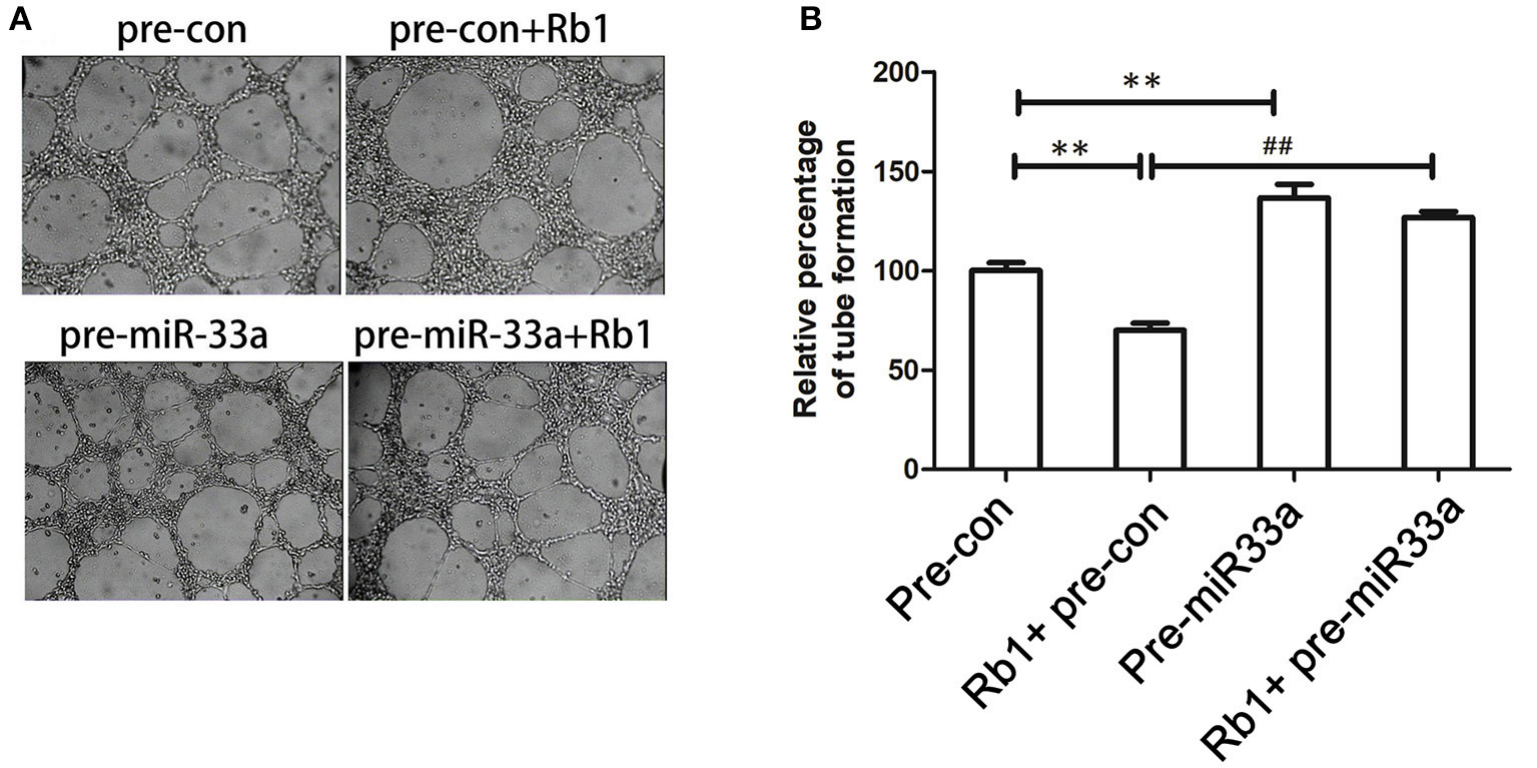
Role of miR-33a in the anti-angiogenic effect of Rb1. **(A)** miR-33a regulates *in vitro* tube formation of HUVECs. Cells transfected with different oligonucleotides including pre-con or pre-miR-33a with or without Rb1 treatment were seeded on Matrigel-coated culture plate and allowed to form vessel-like networks. **(B)** Quantification of tube formation. Each value was expressed as fold of control mean + S.EM. (*n* = 3). ^**^vs. con *p* < 0.01; ^*##*^vs. Rb1 treatment, *p* < 0.01.

We had demonstrated that miR-33a was involved in the regulation of PEDF expression, which was related to angiogenesis. We performed tube formation assay to determine whether miR-33a could modulate angiogenesis *in vitro*. When HUVECs were transfected with pre-miR-33a or pre-control with or without Rb1 treatment, the Rb1-inhibited tube formation was partly abrogated by pre-miR-33a (Figures 3A,B). Meanwhile, pre-miR-33a treatment could increase tube formation as compared with the control (Figures 3A,B). Therefore, miR-33a plays a vital role in the regulation of Rb1-mediated anti-angiogenesis *in vitro*.

### The role of nuclear receptor *PPAR*-γ in *Rb1*-mediated regulation of *miR*-*33a* and *PEDF*

**Figure 4:**
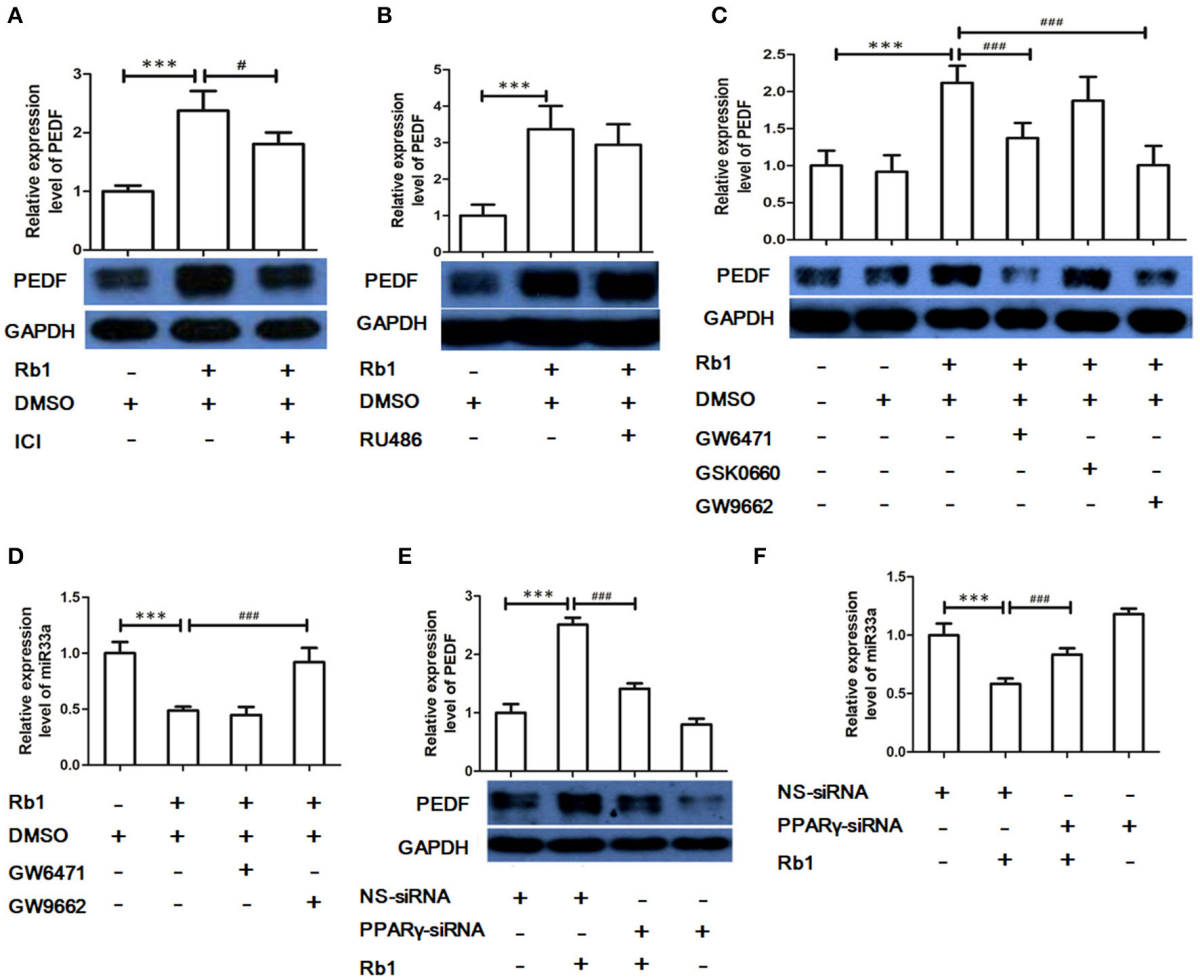
The role of nuclear receptors in Rb1-mediated regulation of miR-33a and PEDF. **(A)** PEDF expression in HUVECs pre-treated with estrogen receptor (ER) antagonist ICI 182,780 (ICI, 20 μM) before Rb1 stimulation. **(B)** PEDF expression in HUVECs pre-treated with Glucocorticoid receptor (GR) antagonist (RU486, 5 μM) before Rb1 stimulation. **(C)** PEDF expression in HUVECs pre-treated with PPARα antagonist (GW6471, 10 μM), PPAR β antagonist (GSK0660, 10 μM), or PPARγ antagonist (GW9662, 10 μM) before Rb1 stimulation. **(D)** Effect of PPARα antagonist (GW6471), PPARγ antagonist (GW9662) on miR expression. **(E)** Effect of PPARα and PPARγ-siRNA on PEDF expression. HUVECs were transiently transfect with treated with PPARα-siRNA (40 nM) and PPARγ-siRNA (40 nM) for 24 h before treatment of Rb1 (10 nM). **(F)** Effect of PPARα and PPARγ-siRNA on miR-33a expression. HUVECs were transiently transfect with treated with PPARα-siRNA (40 nM) and PPARγ-siRNA (40 nM) for 24h before treatment of Rb1 (10 nM). The relative expressions of miRNAs were calculated against U6 RNA using the comparative Ct method (2^−ΔΔCt^). Each value was expressed as fold of control mean + S.E.M (*n* = 3). ^***^p < 0.001 vs. con; ^#^*p* < 0.05, ^*###*^*p* < 0.001 vs. Rb1 treatment alone.

The effects of ginsenosides have been suggested to be through nuclear receptors (Chan et al., 2012; Kwok et al., 2012). To investigate which nuclear receptor was involved Rb1-mediated regulation of miR-33a and PEDF, HUVECs were pre-treated with different pharmacological inhibitors, including GR (RU486), estrogen receptor (ER, ICI 182780), peroxisome proliferator-activated receptor (PPAR)-α (GW6471), PPAR-β (GSK0660), and PPAR-γ (GW9662) antagonist, before Rb1 stimulation. ER, PPAR-α, and PPAR-γ antagonist but not GR or PPAR-β antagonist reversed Rb1-induced PEDF (Figures 4A–C). In addition, GW9662 but not GW6471 could reverse the inhibitory effect of Rb1 on miR-33a expression (Figure 4D). Furthermore, PPAR-γ siRNA was used to explore the role of PPAR-γ in regulating Rb1-increased PEDF expression and reduced miR-33a expression. PPAR-γ knockdown could reduce PEDF expression and increase miR-33a expression (Figures 4E,F), which suggests that the effect of Rb1 on miR-33a and its target gene PEDF expression was PPAR-γ-dependent.

## Discussion

In this study, we found that Rb1 treatment on human endothelial cells, through the interaction with PPAR-γ, decreased miR-33a level and increased PEDF expression, which resulted in the inhibition of angiogenesis. Overexpression of miR-33a significantly reverted the Rb1-induced PEDF expression and anti-angiogenic effect *in vitro*. Moreover, Rb1-mediated PEDF increment and miR-33a reduction were blocked by the pre-incubation with GW9662, an inhibitor of PPAR-γ, as well as PPAR-γ siRNA, which suggests that the action of Rb1 was PPAR-γ-dependent.

Ginseng, a traditional herbal medicine, has been used to treat various diseases for thousands of years. Accumulating evidences have revealed its biological activities of ginsenoside Rb1 in diverse pathological processes, including cardiovascular diseases, neurodegenerative diseases, diabetes, obesity, and cancer, etc. (Sato et al., 1994; Xiong et al., 2010; Cho, 2012; Lee and Kim, 2014). Our previous findings had also revealed that Rb1 exerted anti-angiogenic effects, which may have the therapeutic potential in cardiovascular disease (e.g., atherosclerosis, hypertension, restenosis, etc.), central nervous system diseases (e.g., Alzheimer's disease, stroke, diabetic retinopathy, etc.), chronic inflammatory diseases (e.g., obesity, diabetes, Crohn's disease), and the control cancer development and progression (D'Alessio et al., 2015; Jayakumar et al., 2017; Ramjiawan et al., 2017; Wu et al., 2017).

PEDF can block abnormal neovessels without overt harm to the established retinal vessels even at high doses, which provides the advantages for therapy of angiogenesis-related diseases (Stellmach et al., 2001; Bouck, 2002). In addition to the potent and selective anti-angiogenic function, PEDF has neuroprotective, antioxidant, anti-inflammation, and antithrombotic properties, and has been associated with therapy of atherosclerotic diseases, choroidal neovascularization, cancer, etc. (Takenaka et al., 2008; Rychli et al., 2009; Yamagishi and Matsui, 2010, 2014; Wen et al., 2017).

We had found that Rb1 exerted anti-angiogenic activity via the induction of PEDF, even at relatively low doses (5–20 nM). Also, PEDF was involved in Rb1-mediated inhibition of endothelial tube formation, which was consistent with our previous findings (Leung et al., 2007). Our study had demonstrated that Rb1 (250 nM) could induce both PEDF mRNA and protein expressions (Leung et al., 2007). Here, Rb1 (10 nM) was able to increase PEDF protein expression but without any change on the mRNA level, and the major difference between the present and the previous work is difference in concentration of Rb1. Thus, Rb1 at various concentrations exerted different effects on PEDF expression which contributed to distinct actions. We speculated that post-transcriptional modulation may be involved in this process.

MiRNAs, a novel class of endogenous, small, non-coding RNAs, can regulate gene expression by inhibiting translation and/or degradation of their target genes (Bartel, 2007). Recently, we studied the possible roles of miRNAs in ginsenoside-mediated angiogenesis (Chan et al., 2009, 2013). Expression profiling of various miRNAs in HUVECs confirmed that ginsenoside-Rb1 could modulate miRNA expression. We verified that Rb1 suppressed miR-33a mRNA expression in HUVECs in a time-dependent manner. Furthermore, pre-miR-33a indeed partially reversed the Rb1-increased PEDF expression. We further confirmed that miR-33a reduced PEDF 3′-UTR reporter activity, which suggested that miR-33a could affect the translation of PEDF by targeting its 3′-UTR region. All of these findings supported that Rb1 could promote PEDF protein expression by downregulating miR-33a. This is the first report demonstrated that miR-33a plays a key role in Rb1-mediated PEDF induction and that PEDF is one of the target genes of miR-33a.

MiR-33a was primarily identified as a key regulator of metabolic programs including cholesterol and fatty acid homeostasis (Rayner et al., 2010). It is an intronic miRNA located with the sterol response element-binding protein (SREBP)-2 gene, which codes for transcription factors that coordinate cholesterol, fatty acid synthesis, and HDL metabolism (Marquart et al., 2010). Antagonism of miR-33 upregulates ATP-binding cassette transporter A1 (ABCA1) expression and promotes cholesterol efflux to ApoA-I, a key step in regulating reverse cholesterol transport (Gerin et al., 2010; Rayner et al., 2010). Cholesterol is a structural component of the cell and is indispensable for normal cellular function (Yvan-Charvet et al., 2010). Also, ApoA-I binding protein (AIBP)-targeted cholesterol efflux from ECs could disrupt lipid rafts and inhibit angiogenesis (Fang et al., 2013). Lipid rafts has the ability to support vascular endothelial growth factor (VEGF)-induced dimerization of VEGF receptor 2 (VEGFR2), the major pro-angiogenic receptor in ECs. It was also reported that AIBP removes cholesterol from ECs for reduced lipid raft and VEGFR2 dimerization, which further inhibits VEGFR2 signaling and angiogenesis *in vitro* (Fang and Miller, 2013). Thus, miR-33a reduces cholesterol efflux, but cholesterol efflux is critical for proper angiogenesis (Fang et al., 2013; Sene et al., 2013). However, there is no direct evidence to confirm the role of miR-33a in angiogenesis so far. In order to test the effect of miR-33a on angiogenesis, tube formation assay was performed. Pre-miR-33a transfection could significantly induce the formation of a capillary-like network *in vitro*, which was also able to reverse the inhibitory effects of Rb1 on tubulogenesis. However, miR-33a overexpression could also decrease Rb1-induced PEDF level, which highlights the key role of miR-33a in the PEDF-mediated angiogenic pathway.

Ginsenosides have a four trans-ring steroid-like skeleton structure so they can bind and activate different steroid hormone receptors to regulate gene expression (Attele et al., 1999; Kwok et al., 2012). Intracellular steroid binding proteins could be attractive targets for ginsenosides. We identified that Rb1 acted though PPAR-γ to downregulate miR-27b in 3T3-L1 cells and though PPAR-δ to decrease miR-25 in fibroblast cells (Chan et al., 2012; Kwok et al., 2012). To determine whether steroid hormone receptors are involved in regulation of Rb1 on miR-33a and PEDF, different specific antagonists were used. Western blot results demonstrated that Rb1-increased PEDF expression was abolished by both PPAR-γ and PPAR-α antagonists. However, only PPAR-γ was involved in the Rb1-downregulated miR-33a level. These results highlight the indispensable role of PPAR-γ in Rb1-induced miR-33a and reduced PEDF expression.

It had been suggested that PEDF could interact with PPAR-α for the regulation of angiogenesis and lipid metabolism in hepatocellular carcinoma (Chung et al., 2008). Our previous finding demonstrated that Rb1 was an anti-angiogenic compound in suppressing the formation of endothelial tube-like structures by modulating PEDF via estrogen receptor (ER) (Leung et al., 2007). PEDF could induce apoptosis by activating p53 and PPAR-γ signaling in HUVECs as well (Ho et al., 2008). In the present study, Rb1-induced PEDF expression was mediated by PPAR-γ activation and leading to the anti-angiogenic effect. Recently, multiple findings indicated the association of PPAR-γ with several miRNAs, which were believed to regulate many biological processes through post-transcriptional modulation. MiR-27a could directly target the 3′-UTR of PPAR-γ mRNA in 3T3-L1, thereby inhibiting adipogenesis (Chan et al., 2012). Our current findings suggest that the downregulation of miR-33a in Rb1-exposed HUVECs cells was blocked by the inhibition of PPAR-γ, so several processes may be involved. Specifically, PPAR-γ may bind directly to the miR-33a transcription regulatory sequence or interact with other transcription factors that bind to the miR-33a promoter. On the other hand, Drosha is responsible for the cleavage of pri-miRNAs into pre-miRNAs, and then further processed by Dicer to produce mature miR-33a (Krol et al., 2010). Direct interaction of PPAR-γ with Drosha may thus decrease mature miR-33a biogenesis (Ottaviani et al., 2014).

**Figure 5:**
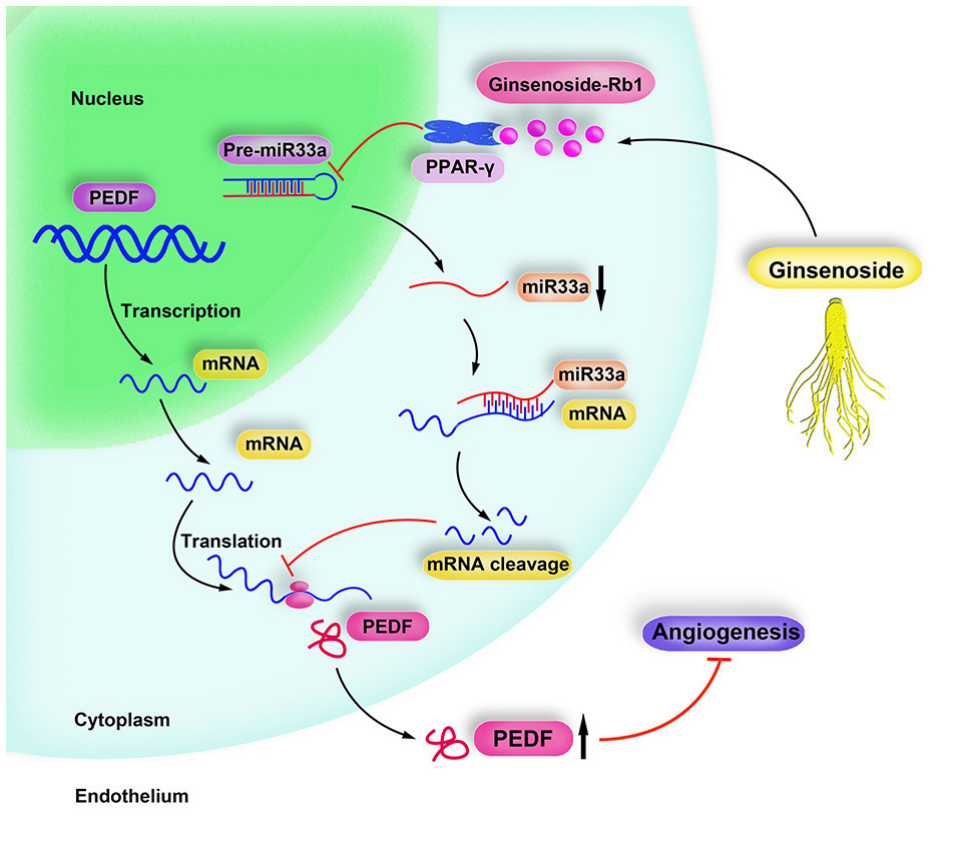
Schematic diagram of Rb1 inhibiting angiogenesis. Ginsenoside Rb1 suppresses miR-33a expression via activating PPARγ, leading to increment of PEDF expression, thereby inhibiting angiogenesis.

In our study, we first elaborated the tight link between miR-33a modulation and function by PPAR-γ signaling. We also provide the direct evidence of Rb1 exerted anti-angiogenic effect which involves the activation of PPAR-γ and downregulation of miR-33, thereby stimulating PEDF. Here, a schematic diagram was drawn to describe the protective role of Rb1 in the inhibition of angiogenesis and the underlying mechanisms (Figure 5), which deepens our understanding of Rb1 in treating ARD.

## Author contributions

HL designed and performed the experiments. XZ performed the experiments. HL and XZ wrote the manuscript. H-HK and P-YP helped with the preformation of the experiments and collection of samples. MD and XL participated in data analyses. ZL contributed to the discussion of the manuscript. RN-SW participated in the design and revision of the manuscript. All authors read and approved the final manuscript.

### Conflict of interest statement

The authors declare that the research was conducted in the absence of any commercial or financial relationships that could be construed as a potential conflict of interest.
